# Loss of the Mitochondrial Fatty Acid β-Oxidation Protein Medium-Chain Acyl-Coenzyme A Dehydrogenase Disrupts Oxidative Phosphorylation Protein Complex Stability and Function

**DOI:** 10.1038/s41598-017-18530-4

**Published:** 2018-01-09

**Authors:** Sze Chern Lim, Makiko Tajika, Masaru Shimura, Kirstyn T. Carey, David A. Stroud, Kei Murayama, Akira Ohtake, Matthew McKenzie

**Affiliations:** 1 Centre for Genetic Diseases, Hudson Institute of Medical Research, 3168 Melbourne, Australia; 2 Department of Molecular and Translational Science, Monash University, 3168 Melbourne, Australia; 3 Department of Metabolism, Chiba Children’s Hospital, 266-0007 Chiba, Japan; 4 Centre for Cancer Research, Hudson Institute of Medical Research, 3168 Melbourne, Australia; 5 Department of Biochemistry and Molecular Biology, Monash Biomedicine Discovery Institute, Monash University, 3800 Melbourne, Australia; 6 Department of Pediatrics, Saitama Medical University, 350-0495 Saitama, Japan

## Abstract

Medium-chain acyl-Coenzyme A dehydrogenase (MCAD) is involved in the initial step of mitochondrial fatty acid β-oxidation (FAO). Loss of function results in MCAD deficiency, a disorder that usually presents in childhood with hypoketotic hypoglycemia, vomiting and lethargy. While the disruption of mitochondrial fatty acid metabolism is the primary metabolic defect, secondary defects in mitochondrial oxidative phosphorylation (OXPHOS) may also contribute to disease pathogenesis. Therefore, we examined OXPHOS activity and stability in MCAD-deficient patient fibroblasts that have no detectable MCAD protein. We found a deficit in mitochondrial oxygen consumption, with reduced steady-state levels of OXPHOS complexes I, III and IV, as well as the OXPHOS supercomplex. To examine the mechanisms involved, we generated an MCAD knockout (KO) using human 143B osteosarcoma cells. These cells also exhibited defects in OXPHOS complex function and steady-state levels, as well as disrupted biogenesis of newly-translated OXPHOS subunits. Overall, our findings suggest that the loss of MCAD is associated with a reduction in steady-state OXPHOS complex levels, resulting in secondary defects in OXPHOS function which may contribute to the pathology of MCAD deficiency.

## Introduction

Medium-chain acyl-CoA dehydrogenase (MCAD, EC 1.3.8.7) is involved in the first reaction of mitochondrial fatty acid β-oxidation (FAO), catalyzing C4 to C12 straight-chain acyl-CoAs^1^. MCAD is translated as a 421 amino acid precursor, which upon import into the mitochondrial matrix, is processed into a mature 396 amino acid (43.6 kDa) protein before forming an enzymatically active ~175 kDa homotetrameric complex^2^.

Loss of MCAD function can result in MCAD deficiency (OMIM: #201450), a disorder affecting approximately 1 in 9,000–15,000 individuals in the Caucasian population^3,4^. Presentation is usually in childhood between the ages of three to 24 months, with patients asymptomatic until a period of prolonged fasting or a common illness triggers symptoms of hypoketotic hypoglycemia, vomiting and lethargy. Hepatopathy, encephalopathy, seizures and coma can be associated with acute episodes of the disease, with a fatal outcome for some patients. A 985A>G transition in *ACADM* has been identified as a common mutation associated with MCAD deficiency in Western Europe, however this mutation has not been identified in the Asian population^5^.

FAO is the primary biochemical pathway for fueling hepatic ketogenesis, a major source of energy once local glycogen stores have been depleted during times of fasting or high energy requirements. MCAD deficiency not only disrupts this process, but also results in the accumulation of fatty acid intermediates such as octanoic and decanoic acids. These intermediates can inhibit mitochondrial oxygen consumption and induce oxidative stress, thereby contributing to the pathogenesis of MCAD deficiency^6–8^. However, some aspects of disease pathology remain unclear, such as the persistent encephalopathy in some patients despite treatment to normalize blood glucose concentrations^9^. One possible pathogenic mechanism may involve the direct effects of MCAD deficiency on mitochondrial oxidative phosphorylation (OXPHOS). MCAD was previously shown to associate with the OXPHOS supercomplex^10^, a high-molecular weight structure that contains OXPHOS complexes I, III and IV^11^. Furthermore, this complex was able to oxidize palmitoyl-CoA and octanoyl-CoA, providing evidence for a physical association between MCAD (as well as other FAO enzymes) with the OXPHOS supercomplex^10^.

These findings support the concept of an additional pathogenic mechanism in MCAD deficiency, whereby the loss of MCAD may directly disrupt the OXPHOS supercomplex, resulting in secondary OXPHOS defects and mitochondrial respiratory dysfunction. To investigate this, we examined the respiratory capacity of the OXPHOS system and the stability of the OXPHOS complexes and supercomplexes. We found that mitochondrial oxygen consumption in MCAD-deficient patient fibroblasts and gene-targeted MCAD ‘knockout’ (KO) cells was reduced compared to controls. We also found a reduction of the steady-state levels of OXPHOS complexes I, III and IV, as well as the OXPHOS supercomplex. Pulse-chase labelling of mtDNA-encoded subunits and *in vitro* mitochondrial import assays of the nuclear-encoded complex IV subunit COX VIa-L revealed defects in OXPHOS complex biogenesis that were associated with the loss of OXPHOS supercomplex stability. These OXPHOS defects resulted in an increased sensitivity to the OXPHOS complex III inhibitor antimycin A, with a concomitant increase in mitochondrial oxidative stress.

Our results suggest that the loss of MCAD is associated with OXPHOS complex instability and dysfunction which may contribute to the pathogenesis of MCAD deficiency.

## Results

### MCAD-deficient patient fibroblasts exhibit defects in mitochondrial respiration and OXPHOS complex steady-state levels

We examined mitochondrial oxygen consumption rates (OCR) in patient fibroblasts to determine whether MCAD-deficiency disrupts mitochondrial respiration. In the presence of glucose, Patient 1 (55%, p < 0.01) and Patient 2 (78%, p < 0.01) fibroblasts both exhibited reduced basal OCR compared to control fibroblasts (Table 1). FCCP-stimulated rates were also significantly reduced in Patient 1 and 2 fibroblasts (44%, p < 0.01 and 56%, p < 0.01 respectively) (Table 1).

**Table 1:** Mitochondrial Oxygen Consumption Rates (OCR) in intact MCAD-deficient patient fibroblasts.

	Mitochondrial Oxygen Consumption Rates pmol/min/20,000 cells (% of Control Values)				
	OCR-Basal	OCR-O	OCR-F	OCR-R	FCCP-stimulated
Control	51 ± 12	19 ± 5	89 ± 24	15 ± 6	74 ± 19
Patient 1 (P1)	28 ± 5* (55%)	11 ± 2* (57%)	42 ± 9* (47%)	9 ± 3* (63%)	32 ± 8* (44%)
Patient 2 (P2)	40 ± 8* (78%)	14 ± 3* (73%)	53 ± 11* (60%)	12 ± 2 (79%)	42 ± 9* (56%)

OCR-O = OCR with oligomycin; OCR-F = OCR with FCCP; OCR-R = OCR with rotenone.

FCCP-stimulated = OCR-F minus OCR-R.

Each condition was performed in at least 14 wells on the same plate.

*p < 0.01.

**Figure 1:**
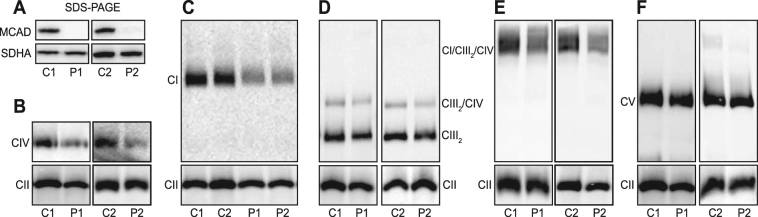
MCAD-deficient patient fibroblasts have reduced steady-state levels of OXPHOS complexes and supercomplexes. Mitochondria were isolated from control (C1 and C2) or MCAD-deficient patient (P1 and P2) fibroblasts for SDS-PAGE and BN-PAGE Western blot analysis. **(A)** No mature MCAD protein is detectable in P1 or P2 patient mitochondria by SDS-PAGE (the OXPHOS complex II subunit SDHA is shown as a loading control). BN-PAGE revealed that the steady-state levels of mature OXPHOS complex IV (detected with an anti-COI antibody) (**B)**, complex I (anti-NDUFA9 antibody) **(C)**, the complex III dimer (CIII_2_) and the CIII_2_/CIV supercomplex (anti-UQCRC1 antibody) **(D)** and the CI/CIII_2_/CIV supercomplex (anti-NDUFA9 antibody) (**E)** were all reduced in P1 and P2 patient mitochondria compared to C1 and C2 controls respectively. **(F)** Steady-state levels of OXPHOS complex V (anti-ATP5A antibody) were no different in P1 and P2 patient mitochondria compared to controls. Mitochondria were solubilised in TX-100 (**B–D,F**) or in digitonin (**E**). Quantitation is relative to the steady-state levels of OXPHOS complex II (anti-SDHA antibody) (n = 3).

These respiration defects were associated with the complete loss of MCAD protein, with no mature MCAD detectable by SDS-PAGE and Western blot analysis (Fig. 1A). Probing for individual OXPHOS subunits in Patient 1 (P1) mitochondria revealed no differences in the steady-state levels of NDUFB8 and NDUFB9 (complex I), SDHA and SDHB (complex II), UQCRC2 (complex III), COII (complex IV) or ATP5A (complex V) (Supplemental Figure S1).

We next used BN-PAGE to examine how the loss of MCAD protein affects the steady-state levels of the individual mature OXPHOS complexes and the OXPHOS supercomplexes (Fig. 1). When solubilised in 1% (v/v) Triton X-100, both Patient 1 (P1) and Patient 2 (P2) fibroblast mitochondria exhibited reduced steady-state levels of monomeric complex IV (P1, 41 ± 16%, p = 0.003; P2, 51 ± 8%, p = 0.001) and monomeric complex I (P1, 62 ± 8%, p = 0.001; P2, 57 ± 8%, p = 0.001) compared to control fibroblasts (Fig. 1B and C, Supplemental Table S2). Steady-state levels of the complex III dimer (CIII_2_) (P1, 79 ± 8%, p = 0.01; P2, 82 ± 4%, p = 0.001) and the complex CIII_2_/CIV supercomplex (P1, 78 ± 10%, p = 0.002; P2, 76 ± 13%, p = 0.03) were also reduced in patient P1 and P2 mitochondria compared to controls (Fig. 1D). Solubilisation of mitochondria in 1% (w/v) digitonin also revealed that the steady-state levels of the OXPHOS CI/CIII_2_/CIV supercomplex were reduced in P1 and P2 patient fibroblasts compared to controls (P1, 68 ± 2%, p < 0.001; P2, 74 ± 5%, p = 0.001) (Fig. 1E). In contrast, the levels of complex V were not different between patients (P1, p = 0.8; P2, p = 0.5) and controls (Fig. 1F).

### Deletion of MCAD in 143B cells replicates the OXPHOS defects observed in MCAD-deficient patient fibroblasts

We edited the *ACADM* gene in 143B cells using a lentiviral-based CRISPR/Cas9 system to confirm that the OXPHOS defects observed in patient fibroblast mitochondria were due to MCAD deficiency. We selected the 143B cell line as it grows readily in culture, allowing us to produce large numbers of cells for detailed biochemical analyses.

**Figure 2:**
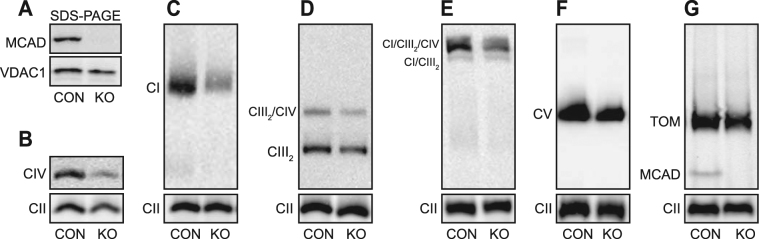
143B MCAD knockout (KO) cells also exhibit reduced steady-state levels of OXPHOS complexes and supercomplexes. The *ACADM* gene was edited in 143B cells using lentiviral-based CRISPR/Cas9. Mitochondria were isolated from 143B control cells (CON) or a 143B MCAD knockout clone (KO) for SDS-PAGE and BN-PAGE Western blot analysis. **(A)** No mature MCAD protein is detectable in KO mitochondria by SDS-PAGE (the mitochondrial protein VDAC1 is shown as a loading control). BN-PAGE revealed that the steady-state levels of mature OXPHOS complex IV (detected with an anti-COI antibody) **(B)**, complex I (anti-NDUFA9 antibody) (**C)**, the complex III dimer (CIII_2_) and the CIII_2_/CIV supercomplex (anti-UQCRC1 antibody) **(D**) and the CI/CIII_2_/CIV supercomplex (anti-NDUFA9 antibody) (**E**) were all significantly reduced in KO mitochondria compared to the control (CON). Steady-state levels of OXPHOS complex V (anti-ATP5A antibody) **(F**) and the Translocase of the Outer Mitochondrial Membrane (anti-TOMM40 antibody) **(G**) were not different in KO mitochondria compared to the control (CON). **(G)** The homotetrameric MCAD complex is detectable in CON, but not MCAD KO mitochondria. Mitochondria were solubilised in TX-100 (**B–D**) or in digitonin (**E–G**). Quantitation is relative to the steady-state levels of OXPHOS complex II (anti-SDHA antibody) (n = 3).

Following transduction of a CRISPR/Cas9 construct containing an RNA guide targeting exon 2 of *ACADM*, twelve 143B single-cell clones were identified. Of these, eight clones had undetectable levels of MCAD protein (data not shown). Genotyping of clone C2 identified an insertion and/or deletion in the *ACADM* alleles (Supplemental Figure S2), resulting in the loss of MCAD protein expression (Fig. 2A). This clone was used in all subsequent experiments and designated the MCAD KO (‘knockout’).

Similar to MCAD-deficient patient fibroblast mitochondria, 143B MCAD KO mitochondria exhibited reduced steady-state levels of complex IV (45 ± 8%, p < 0.001) (Fig. 2B), complex I (53 ± 8%, p = 0.001) (Fig. 2C), the complex III dimer (CIII_2_) (75 ± 8%, p = 0.006) (Fig. 2D), the complex CIII_2_/CIV supercomplex (68 ± 13%, p = 0.01) (Fig. 2D) and the CI/CIII_2_/CIV supercomplex (69 ± 13%, p = 0.01) (Fig. 2E) compared to 143B control mitochondria (CON) (Supplemental Table S2). Levels of complex V (p = 0.16) (Fig. 2F), as well as the translocase of the outer mitochondrial membrane (TOM) complex (p = 0.21) (Fig. 2G), were not affected in MCAD KO mitochondria. In 143B control mitochondria, the homotetrameric ~175 kDa MCAD complex was clearly detected by BN-PAGE and Western blotting (Fig. 2G). This is in contrast to human fibroblast mitochondria, where MCAD expression was too low to detect this native complex. As would be expected, the MCAD complex is absent in MCAD KO mitochondria (Fig. 2G).

**Figure 3:**
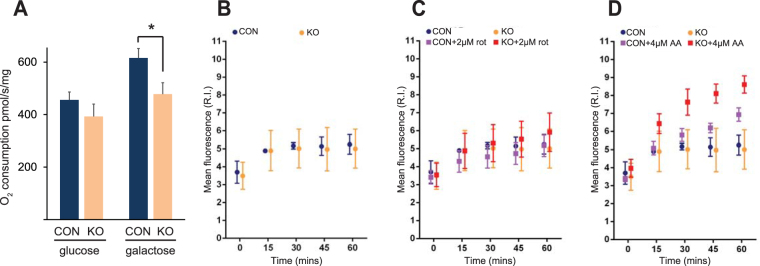
143B MCAD knockout (KO) cells exhibit defects in mitochondrial respiration and are more sensitive to OXPHOS inhibitor-induced oxidative stress. (**A**) Mitochondrial oxygen consumption in MCAD knockout (KO) cells was not significantly different to control (CON) cells in the presence of glucose. However, oxygen consumption was significantly lower in MCAD KO cells in the presence of galactose. Data is mean ± s.d., n = 3. *p < 0.05. Mitochondrial reactive oxygen species generation was assessed in live cells using the superoxide probe MitoSOX^TM^. There was no difference in mitochondrial superoxide generation between CON and MCAD KO cells over a 60 min time course in either untreated conditions (**B**) or with the addition of the OXPHOS complex I inhibitor rotenone (**C)**. **(D**) Mitochondrial superoxide generation was significantly greater in MCAD KO cells compared to CON cells after 30 min (p = 0.001), 45 min (p = 0.0005) and 60 min (p = 0.0034) in the presence of the OXPHOS complex III inhibitor antimycin A. Data is mean ± s.d., n = 5.

We next examined the effects of MCAD deletion on mitochondrial respiration, and found no difference in mitochondrial oxygen consumption between control cells and MCAD KO cells in the presence of glucose (p = 0.12, Fig. 3A). Galactose is metabolized slowly to glucose-1-phosphate, resulting in low glycolytic ATP generation and the stimulation of oxidative phosphorylation^12^. In the presence of this sugar, mitochondrial oxygen consumption was lower in MCAD KO cells compared to the control (78 ± 7% residual activity, p = 0.01) (Fig. 3A).

### Antimycin A augments reactive oxygen species production in MCAD knockout cells

Elevated oxidative stress has been suggested as a factor that contributes to the pathogenesis of MCAD deficiency, with increased levels of both oxidized proteins and phospholipids detected in MCAD-deficient patient samples^8,13^. We examined the generation of mitochondrial reactive oxygen species in live MCAD KO cells using the mitochondria-specific superoxide probe MitoSOX^TM^. There was no difference in the amount of mitochondrial superoxide generated over a 60 min time course between untreated control and MCAD KO cells (p = 0.99) (Fig. 3B). Similarly, in the presence of the OXPHOS complex I inhibitor rotenone (rot), there was no difference in superoxide generated between control and MCAD KO cells (p = 0.54) (Fig. 3C). However, in the presence of the OXPHOS complex III inhibitor antimycin A, mitochondrial superoxide levels were higher in MCAD KO cells compared to control cells after 30 min (p = 0.001), 45 min (p = 0.0005) and 60 min (p = 0.0034) (Fig. 3D). These findings suggest that MCAD KO mitochondria are more sensitive to the inhibition of electron transfer through complex III, with a concomitant increase in free radical generation.

### Incorporation of newly-translated mtDNA-encoded subunits into the OXPHOS complexes and the OXPHOS supercomplex is disrupted in MCAD KO cells

**Figure 4:**
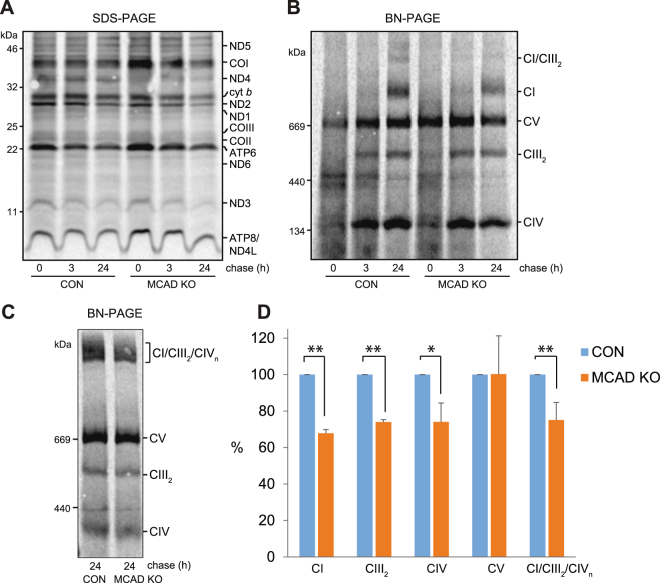
Incorporation of newly-translated mtDNA-encoded subunits into the OXPHOS complexes and the OXPHOS supercomplex is disrupted in MCAD knockout cells. MtDNA-encoded-proteins were radiolabeled in the presence of cycloheximide and chased for 0, 3 or 24 h. (**A**) SDS-PAGE showing similar amounts of newly-translated complex I (ND1, ND2, ND3, ND4L, ND5 and ND6), complex III (cyt *b*), complex IV (COI, COII and COIII) and complex V (ATP6 and ATP8) subunits in both 143B control (CON) and 143B MCAD knockout (KO) mitochondria. (**B**) BN-PAGE analysis of mitochondria solubilised in TX-100 shows that the amount of newly-translated mtDNA-encoded proteins incorporated into complex I (CI), the complex III dimer (CIII_2_) and complex IV (CIV) after 24 h chase is less in MCAD KO mitochondria than CON mitochondria. Levels of complex V were not different (**C**) BN-PAGE following solubilisation in digitonin. Reduced levels of newly-translated subunits in the CI/CIII_2_/CIV supercomplex are evident in MCAD KO mitochondria compared to control (CON). **(D)** Quantitation of OXPHOS complex and supercomplex levels. Data is mean ± s.d., n = 3. *p < 0.05, **p < 0.01.

We next examined whether the reduction in steady-state OXPHOS complexes levels in MCAD KO cells is due to defects in OXPHOS complex biogenesis. MtDNA encoded-protein subunits were radiolabeled, followed by mitochondrial isolation and SDS-PAGE analysis (Fig. 4A). Subunits of complex I (ND1, ND2, ND3, ND4L, ND5 and ND6), complex III (cyt *b*), complex IV (COI, COII and COIII) and complex V (ATP6 and ATP8) were detected at similar levels in both control and MCAD KO cells following a 2 h pulse (Fig. 4A). Next, assembly of newly-translated mtDNA-encoded proteins into mature OXPHOS complexes was analyzed using a pulse-chase and BN-PAGE (Fig. 4B). The amount of newly-translated subunits incorporated into complex I (68 ± 2%, p < 0.001), Complex III_2_, (74 ± 1%, p < 0.001) and Complex IV (74 ± 10%, p = 0.01) at the 24 h chase time point was less in MCAD KO cells than in control cells, whereas there was no difference in complex V assembly (p = 0.98) (Fig. 4B and D, Supplemental Table S3). Solubilisation of mitochondria in digitonin before BN-PAGE analysis also revealed reduced levels of newly-translated subunits in the CI/CIII_2_/CIV supercomplex in MCAD KO cells (75 ± 10%, p = 0.002) (Fig. 4C and D).

**Figure 5:**
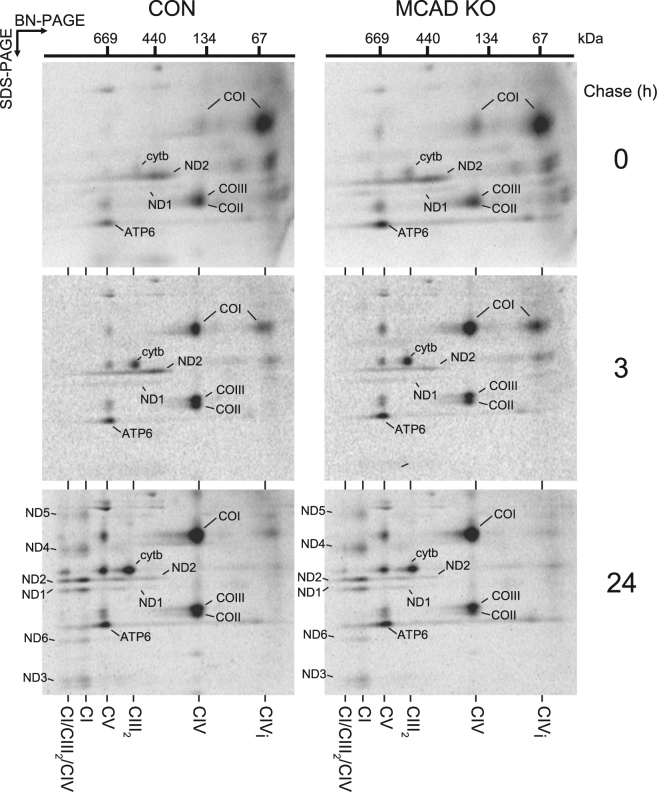
The amount of newly-translated mtDNA-encoded subunits incorporated into the OXPHOS supercomplex is reduced in MCAD knockout mitochondria. MtDNA-encoded proteins were radiolabeled in both 143B control (CON) and 143B MCAD knockout (KO) cells in the presence of cycloheximide and chased for 0, 3 or 24 h. Mitochondria were then isolated for two dimensional (2D) native-PAGE analysis. OXPHOS complex subunits are identified according to^14,38^. Following 24 h chase, the incorporation of subunits ND1, ND2, ND3, ND4, ND5 and ND6 (complex I), Cyt *b* (complex III) and COI, COII and COIII (complex IV) into the CI/CIII_2_/CIV supercomplex is reduced in MCAD KO mitochondria compared to the control (CON) (p = 0.001).

We next performed pulse-chase radiolabeling followed by 2-dimensional (2D)-native PAGE to determine how the assembly of individual mtDNA-encoded subunits into the OXPHOS complexes is affected in MCAD KO cells (Fig. 5). Following the 2 h pulse (0 h chase), the complex I subunits ND1 and ND2 assembled into a ~460 kDa intermediate complex, cyt *b* was detected in the complex III homodimer (CIII_2_), COI was detected in a ~70 kDa complex IV intermediate (CIV_i_) and mature complex IV (CIV) with subunits COII and COIII, and ATP6 was found in mature CV (CV) in both control and MCAD KO mitochondria (Fig. 5, top panels). Some cyt *b*, COI, COII and COIII were also be detected in the ~670 kDa CIII_2_/CIV dimer (which resolves at the same size as ATP6 in mature CV). After 3 h chase, both ND1 and ND2 were detectable in intermediate assemblies ranging from ~460 kDa to ~830 kDa in both control and MCAD KO mitochondria, while cyt *b*, COI, COII, COIII and ATP6 further assembled into their respective mature complexes (Fig. 5, middle panels). Following 24 h chase, the complex I subunits ND1, ND2, ND3, ND4, ND5 and ND6 all assembled into mature complex I (CI) in both control and MCAD KO mitochondria (Fig. 5, bottom panels). These complex I subunits, along with cyt *b*, COI, COII and COIII, also assembled into the CI/CIII_2_/CIV supercomplex in control mitochondria following 24 h chase (Fig. 5, bottom left panel). However, the presence of these subunits in the CI/CIII_2_/CIV supercomplex in MCAD KO mitochondria was reduced to 54 ± 8% (p = 0.001) of control values (Fig. 5, bottom right panel, Supplemental Table S3).

### The assembly of nuclear-encoded subunits during the latter stages of complex IV biogenesis is disrupted in MCAD KO mitochondria

**Figure 6:**
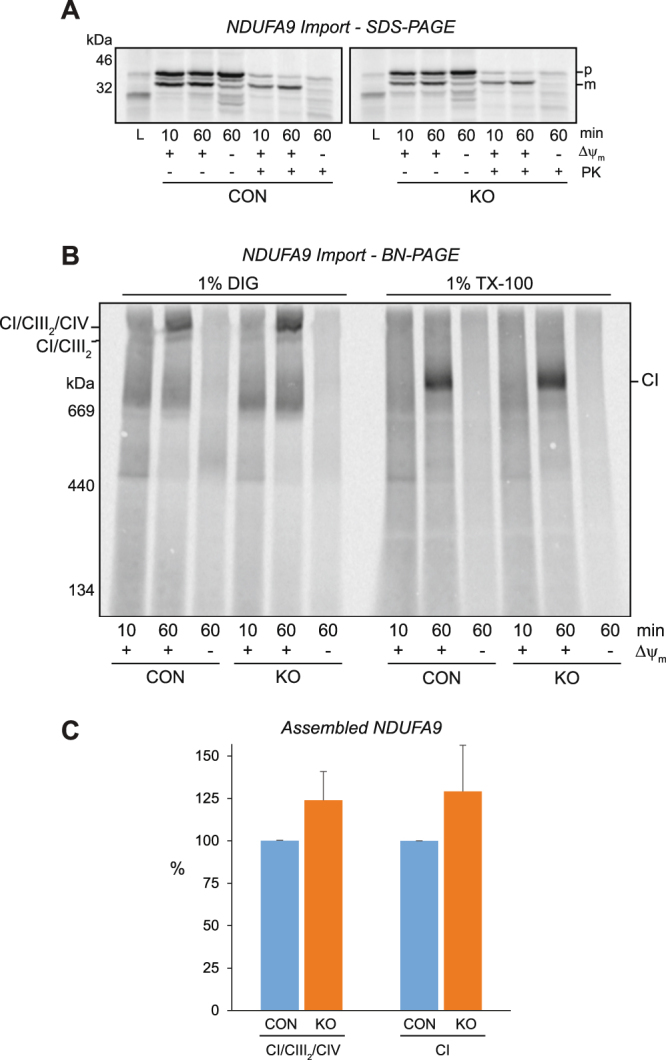
The nuclear-encoded subunit NDUFA9 is imported and assembled efficiently into OXPHOS complex I in 143B MCAD knockout mitochondria. NDUFA9 was radiolabeled by *in vitro* transcription/translation, followed by incubation for 10 and 60 min with isolated 143B control (CON) or 143B MCAD knockout (KO) mitochondria. **(A)** SDS-PAGE showing NDUFA9 in its precursor (p) form and as a proteinase K (PK) resistant mature (m) form. NDUFA9 is imported efficiently into both CON and MCAD KO mitochondria in a mitochondrial membrane potential (Δψ_m_) dependent manner. **(B)** BN-PAGE showing the assembly of NDUFA9 into the CI/CIII_2_ and CI/CIII_2_/CIV supercomplexes (following solubilisation in digitonin, left) or mature complex I (CI, following solubilisation in TX-100, right). **(C)** Quantitation of NDUFA9 assembly after 60 min of import. There was no difference in the amount of NDUFA9 assembled into the CI/CIII_2_/CIV supercomplex (p = 0.07) or complex I (p = 0.14).

As the biogenesis of mtDNA-encoded subunits is altered in MCAD KO cells, we next investigated whether the assembly of nuclear-encoded OXPHOS subunits is also affected. As OXPHOS complexes I and IV exhibit the largest decrease in steady-state levels in both MCAD-deficient patient fibroblasts and MCAD KO cells, we examined the assembly profiles of subunits from these two complexes, NDUFA9 and COX VIa-L. The complex I subunit NDUFA9 assembles into a ~830 kDa late-stage intermediate before integrating into the ~980 kDa holocomplex I^14,15^. NDUFA9 was radiolabeled by *in vitro* transcription/translation using rabbit reticulocyte lysate in the presence of ^35^S-methionine, followed by incubation for 10 and 60 min with isolated control or MCAD KO mitochondria (Fig. 6). Following import, a proteinase K (PK)-resistant mature form of NDUFA9 (m) was detectable at similar levels in both control and MCAD KO mitochondria, indicating normal import and processing of its precursor form (p) (Fig. 6A).

The assembly of newly-imported NDUFA9 into mature OXPHOS complexes was then analyzed by BN-PAGE (Fig. 6). After 60 min of import, solubilisation of mitochondria in digitonin revealed that NDUFA9 assembled in a membrane potential (Δψ_m_) dependent manner into the CI/CIII_2_ and CI/CIII_2_/CIV supercomplexes (Fig. 6B). There was no difference in the amount of NDUFA9 detected in control and MCAD KO mitochondria after this import time (p = 0.07) (Fig. 6B and C, Supplemental Table S3). Similarly, solubilisation of mitochondria in TX-100 showed no difference in the amount of NDUFA9 assembled into mature complex I after 60 min of import (p = 0.14) (Fig. 6B and C).

**Figure 7:**
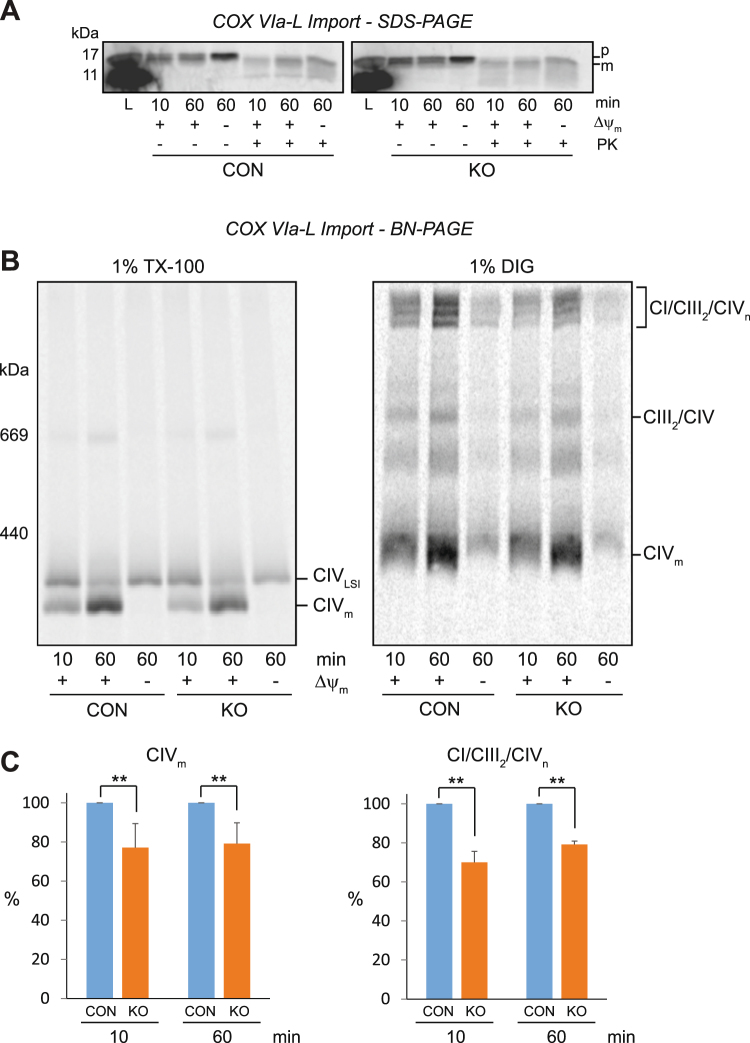
The assembly of the nuclear-encoded subunit COX VIa-L into mature complex IV and the OXPHOS supercomplexes is reduced in 143B MCAD knockout mitochondria. COX VIa-L was radiolabeled by *in vitro* transcription/translation, followed by incubation for 10 and 60 min with isolated 143B control (CON) or 143B MCAD knockout (KO) mitochondria. **(A)** SDS-PAGE showing COX VIa-L in its precursor (p) form and as a proteinase K (PK) resistant mature (m) form. COX VIa-L is imported efficiently into both CON and MCAD KO mitochondria. **(B)** BN-PAGE showing the assembly of COX VIa-L into the late-stage intermediate (CIV_LSI_) and mature complex IV (CIV_m_) [following solubilisation in 1% (v/v) TX-100, left] or the CI/CIII_2_/CIV_n_ supercomplexes [following solubilisation in 1% (w/v) digitonin, right]. The amount of newly-translated COX VIa-L assembled into CIV_m_ and the CI/CIII_2_/CIV_n_ supercomplexes was significantly less in MCAD KO mitochondria compared to CON mitochondria after import times of both 10 and 60 min. **(C)** Quantitation of assembled COX VIa-L into CIV_m_ (n = 4) and the CI/CIII_2_/CIV_n_ supercomplexes (n = 3). Data is mean ± s.d. **p < 0.01.

We also examined the biogenesis of the complex IV subunit COX VIa-L by *in vitro* mitochondrial import (Fig. 7). Similar to the import of NDUFA9, COX VIa-L was imported efficiently into both control and MCAD KO mitochondria, as shown by the presence of a mature, PK resistant form after 60 min of import (Fig. 7A). We previously showed that COX VIa-L initially assembles into a ~250 kDa late stage intermediate (LSI) complex before being integrated into the ~200 kDa holocomplex IV^16^. Following import of COX VIa-L, mitochondria were solubilised in TX-100 for BN-PAGE analysis (Fig. 7B). COX VIa-L assembled into both the LSI intermediate complex (CIV_LSI_) and mature CIV (CIV_m_) in both control and MCAD KO mitochondria. However, the amount of newly-translated COX VIa-L that assembled into mature CIV (CIV_m_) was less in MCAD KO mitochondria compared to control mitochondria after import times of both 10 min (77 ± 12%, p = 0.01) and 60 min (79 ± 11%, p = 0.01) (Fig. 7B and C, Supplemental Table S3).

Mitochondria were also solubilised in digitonin to resolve the OXPHOS supercomplexes on BN-PAGE. COX VIa-L assembled into numerous high molecular weight forms of the OXPHOS supercomplex (CI/CIII_2_/CIV_n_) in both control and MCAD KO mitochondria. However, this assembly was less in MCAD KO mitochondria compared to control mitochondria after import times of both 10 min (70 ± 6%, p = 0.001) and 60 min (79 ± 2%, p < 0.001) (Fig. 7B and C, Supplemental Table S3). These findings suggest a defect in the assembly of COX VIa-L into complex IV and the OXPHOS supercomplex in MCAD KO mitochondria.

### High molecular weight complexes containing MCAD are detectable by native gel electrophoresis

**Figure 8:**
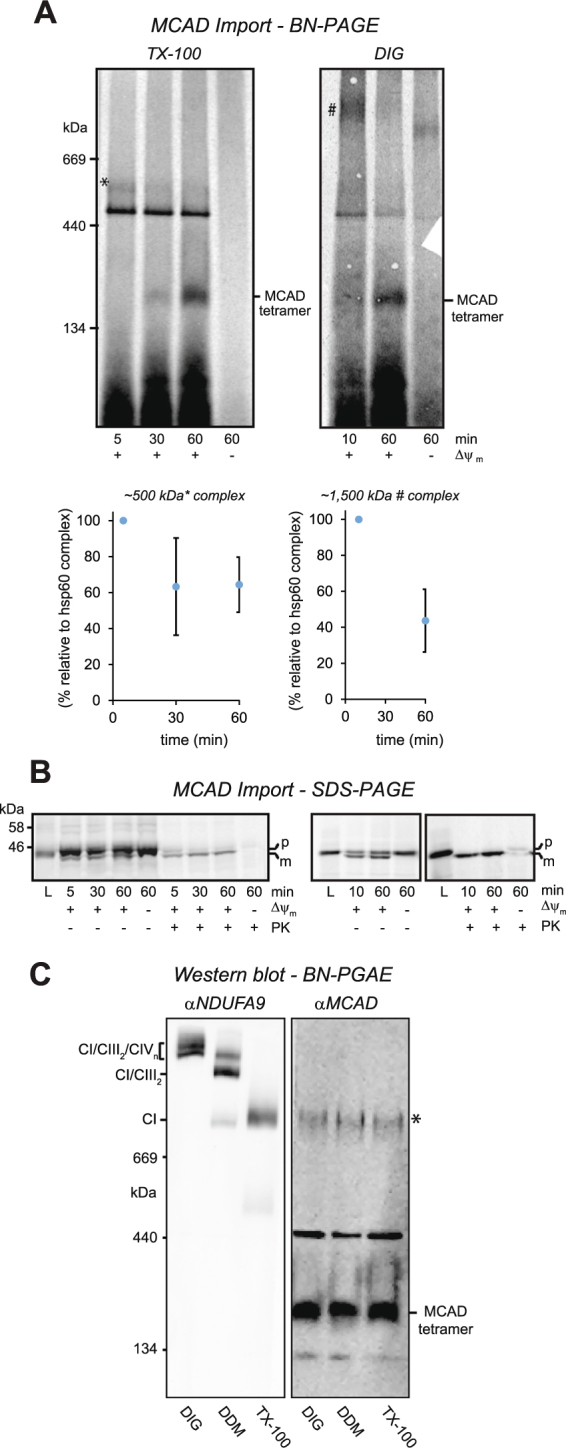
High molecular weight complexes containing MCAD are detectable by BN-PAGE. **(A**) MCAD was radiolabeled by *in vitro* transcription/translation, followed by incubation for 5, 10, 30 and 60 min with isolated 143B control mitochondria. Mitochondria were solubilised in 1% (v/v) TX-100 or 1% (w/v) digitonin, followed by BN-PAGE analysis. MCAD can be detected in complexes of ~175 kDa (MCAD homotetramer) and ~450 kDa (mitochondrial Hsp60 complex), as well as unknown complexes of ~500 kDa (marked *) and ~1,500 kDa (marked #). The intensities of both the ~500 kDa (*) and ~1,500 kDa (#) MCAD-containing complexes (relative to the ~450 kDa mitochondrial Hsp60 complex) was reduced over the 60 min import time course (p = 0.016 and 0.005 respectively). **(B)** SDS-PAGE showing MCAD in its precursor (p) form and as a proteinase K (PK) resistant mature (m) form. MCAD is imported in a mitochondrial membrane potential (Δψ_m_) dependent manner, with the levels of mature MCAD protein (m) similar at each time point for both the TX-100 and DIG experiments. **(C)** BN-PAGE and Western blot analysis of HepG2 cell mitochondria solubilised in 1% (w/v) digitonin (DIG), 0.2% (w/v) dodecyl maltoside (DDM) or 1% (v/v) TX-100. Anti-NDUFA9 antibodies detect monomeric complex I and the CI/CIII_2_/CIV_n_ and CI/CIII_2_ OXPHOS supercomplexes, while anti-MCAD antibodies detect MCAD in complexes of ~130 kDa, ~175 kDa (homotetramer), ~450 kDa (mitochondrial Hsp60 complex) and ~1,000 kDa (*).

To investigate how MCAD influences the steady-state levels of the OXPHOS complexes and supercomplexes, we examined whether MCAD-containing complexes could be detected using BN-PAGE. *In vitro* mitochondrial import assays were performed with radiolabeled MCAD and isolated 143B control mitochondria over a time course of 60 min. Mitochondria were then solubilised in either TX-100 or digitonin (DIG), before being resolved by BN-PAGE (Fig. 8A). After 60 min, the ~175 kDa MCAD homotetramer was detected when mitochondria were solubilised in either TX-100 or DIG. Some newly-imported MCAD also associated with the mitochondria chaperonin Hsp60 complex, which resolves as a tight ~450 kDa band^3^. In addition, an MCAD-containing complex of ~500 kDa (marked *) was resolved following TX-100 solubilisation. This ~500 kDa complex decreased in intensity to 64 ± 15% (p = 0.016) of its original value after 60 min (Fig. 8A, Supplemental Table S3). A ~1,500 kDa MCAD-containing complex was also resolved following DIG solubilisation (marked #), reducing in intensity to 44 ± 17% (p = 0.005) over the 60 min import time course (Fig. 8A, Supplemental Table S3).

Analysis of import by SDS-PAGE revealed that the amount of mature (m) MCAD protein imported into isolated mitochondria was similar at each time point for both the TX-100 and DIG experiments (Fig. 8B), even though the intensities of the ~500 and ~1,500 kDa complexes changed over the same time course.

Following BN-PAGE and Western blotting of 143B control mitochondria, anti-MCAD antibodies were able to detect the ~175 kDa MCAD homotetramer, but no other steady-state MCAD-containing complexes (Fig. 2G). Consequently, we examined mitochondria isolated from HepG2 cells, as the hepatic lineage of these cells predicts higher MCAD expression compared to 143B cells. HepG2 mitochondria were solubilised in either digitonin, dodecyl maltoside or TX-100, separated by BN-PAGE and probed with antibodies against the complex I subunit NDUFA9 or MCAD (Fig. 8C). Anti-NDUFA9 antibodies detected complex I in distinct profiles that were dependent on the detergent used for solubilisation: in DIG, NDUFA9 was detected in the OXPHOS supercomplexes containing CIV (CI/CIII_2_/CIV_n_); in DDM, NDUFA9 was detected in the CI/CIII_2_/CIV and CI/CIII_2_ supercomplexes; and in TX-100, NDUFA9 was found in monomeric complex I (and also to a lesser degree in DDM) (Fig. 8C). Following BN-PAGE and Western blotting, anti-MCAD antibodies detected four distinct steady-state complexes with sizes of ~130 kDa, ~175 kDa, ~450 kDa and ~1,000 kDa (Fig. 8C). Notably, these complexes had exactly the same profile regardless of the detergent used for solubilisation.

**Figure 9:**
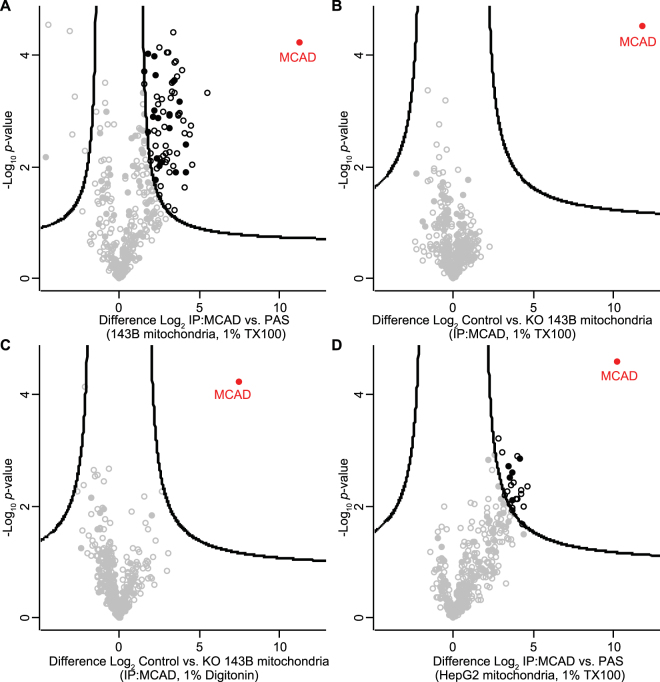
Identification of MCAD interacting proteins. **(A)** Co-immunoprecipitation (Co-IP) and mass-spectrometry analysis of 143B cell mitochondria. Crude mitochondria were solubilised in 1% (v/v) Triton X-100 and proteins incubated with Protein A-sepharose coupled MCAD antibodies or protein A-sepharose alone. Eluates were analyzed by label-free quantitative mass-spectrometry (LFQ). Log2 LFQ intensities were submitted to a modified two-sided two-sample *t*-test with significance determined through permutation-based false discovery rate (FDR) statistics. Closed circles, mitochondrial protein; empty circles, non-mitochondrial protein; black, proteins with <1% FDR. **(B)** As for (A) using either control 143B cell mitochondria or mitochondria from 143B MCAD knockout (KO) cells solubilised in 1% (v/v) Triton X-100, bound to Protein A-sepharose coupled MCAD antibodies in both cases. **(C)** As for (B) using either control 143B mitochondria or mitochondria from 143B MCAD knockout (KO) cells solubilised in 1% (w/v) digitonin, bound to Protein A-sepharose coupled MCAD antibodies in both cases. **(D)** As for (A) using HepG2 control cell mitochondria and 1% (w/v) digitonin, bound to Protein A-sepharose coupled MCAD antibodies or protein A-sepharose alone.

To identify potential interacting partners of MCAD, we performed co-immunoprecipitation (Co-IP) analysis of detergent-solubilised mitochondria followed by quantitative mass spectrometry. Co-IPs were performed in triplicate using Protein A-sepharose bound anti-MCAD antibodies as bait, or with Protein A-sepharose alone as a negative control. Following solubilisation of 143B control mitochondria in TX-100, MCAD was significantly enriched following immunoprecipitation as expected (Fig. 9A). A number of other mitochondrial and cellular proteins were also significantly enriched (Supplemental Table S1). To determine the specificity of their interaction with MCAD, we repeated the experiment using Protein A-sepharose bound anti-MCAD antibodies to Co-IP proteins from either 143B control or 143B MCAD KO mitochondria (Fig. 9B). As can be seen, no proteins strongly interacted (>4 fold enrichment) with MCAD. We also performed the Co-IP using digitonin to maintain higher order interactions (Fig. 9C). Similarly, MCAD was significantly enriched in the absence of any other strong interactors (Supplemental Table S1).

As native MCAD complexes are detected in HepG2 mitochondria (Fig. 8B), we also performed Co-IP/quantitative mass spectrometry using digitonin solubilised mitochondria from this cell type, bound to either Protein A-sepharose coupled anti-MCAD antibodies or Protein A-sepharose alone (Fig. 9D). MCAD was again significantly immunoprecipitated, with other mitochondrial and cellular proteins also significantly enriched (Supplemental Table S1). However, most of these proteins were enriched in the comparable 143B experiment (Fig. 9A), but lost upon comparison with the 143B MCAD knockout (Fig. 9B), suggesting they are binding non-specifically to the MCAD antibodies. Notably, in all cases we did not detect any respiratory chain subunits, in particular those found in the CI/CIII_2_/CIV supercomplex, to be significantly enriched with MCAD (Supplemental Table S1).

## Discussion

Mitochondrial fatty acid β-oxidation (FAO) and oxidative phosphorylation (OXPHOS) are important biochemical processes that metabolize fats and sugars to generate ATP for the cell. FAO and OXPHOS are tightly linked biochemically, with FAO providing reducing equivalents to the OXPHOS pathway in two ways. In the first dehydrogenase step of FAO, electrons are transferred via the Electron transfer flavoprotein and the Electron transfer flavoprotein dehydrogenase to reduce ubiquinone, while the third step of FAO (a second dehydrogenase reaction) generates NADH for oxidation by OXPHOS complex I.

In addition to these crucial biochemical interactions, various studies have shown that FAO and OXPHOS proteins physically interact. OXPHOS complex I can bind several different NAD-coupled dehydrogenases, including the FAO protein L-3-hydroxyacyl-CoA dehydrogenase^17^, whereas the Electron transfer flavoprotein has been identified in purified complexes with OXPHOS complex III^18^. More recently, the FAO proteins Very-long chain acyl-CoA dehydrogenase, Electron transfer flavoprotein, Mitochondrial trifunctional protein and MCAD were shown to co-migrate with monomeric complex I and the CI/CIII_2_/CIV OXPHOS supercomplex by both BN-PAGE and sucrose density gradients^10^. Furthermore, palmitoyl-CoA was completely oxidized by the sucrose gradient fractions that contained these complexes, suggesting the existence of a metabolically active FAO-OXPHOS super-structure that can completely oxidize fatty acids.

Interestingly, it has long been known that patients with primary FAO deficiencies can have associated secondary defects in OXPHOS enzyme activity. Patients with Long-chain hydroxyacyl-CoA dehydrogenase deficiency can exhibit secondary OXPHOS complex I defects^19,20^. Furthermore, of the 23 patients with Enoyl-CoA hydratase (ECHS1) deficiency described to date, six have significant defects in OXPHOS enzyme activity^21^. While it has long been thought that these secondary OXPHOS defects are solely due to the toxic accumulation of fatty-acyl CoA intermediates^18^, the pathology involved may be more complex than first realized. Indeed, the growing evidence for FAO-OXPHOS protein complexes suggests that the loss of FAO protein expression may disrupt crucial FAO-OXPHOS protein interactions, resulting in the subsequent destabilization and loss of the OXPHOS complexes. This may be the case in some ECHS1-deficient patients, with decreased steady-state levels of OXPHOS complex I, III and IV observed^22^.

Our findings here also suggest that the accumulation of fatty acid intermediates in MCAD deficiency is not the only cause of secondary OXPHOS dysfunction. In both MCAD-deficient patient cells and MCAD KO cells, the loss of MCAD protein expression resulted in reduced steady-state levels of OXPHOS complexes I, III and IV, as well as those of the OXPHOS supercomplex. Interestingly, the levels of the individual OXPHOS complex subunits that we examined were not significantly altered, even though their mature OXPHOS complexes were reduced. We have observed this type of subunit stability previously, with the complex I subunits NDUFB6, NDUFB8 and NDUFB9 remaining intact, even when mature complex I is not assembled^23^.

Our mtDNA-encoded protein assembly assay showed that the amount of newly-translated OXPHOS subunits that were incorporated into the OXPHOS complexes and the OXPHOS supercomplex during the early stages of their assembly were unaffected in MCAD-deficient mitochondria. However, after a 24 h chase period, the amount of these subunits found in the mature OXPHOS complexes and the supercomplex were reduced. In conjunction with our BN-PAGE Western blot data, these findings suggest that the loss of MCAD causes the destabilization of OXPHOS complexes I, III and IV (as opposed to a defect in their assembly), with an associated loss of the OXPHOS supercomplex. In support of this, we found that the assembly of NDUFA9 into mature complex I and the OXPHOS supercomplexes was normal in MCAD KO mitochondria. Although our *in vitro* import assays of COX VIa-L did reveal a late-stage defect in its biogenesis, this may also be due to the destabilization of complex IV, which turns over much faster than complex I^24^.

The reduced supercomplex stability detected is similar to that observed in cells harboring pathogenic OXPHOS subunit gene mutations^25,26^ or in Barth Syndrome patient cells, which have mitochondrial cardiolipin remodeling defects due to mutations in *TAZ* ^27^. Interestingly, in MCAD-deficient cells, complexes I and IV were particularly affected, with the steady-state levels of complex III reduced to a lesser degree. This may be due to the increased stability of complex III in its homodimeric form (CIII_2_), whereas monomeric complex I and IV are more unstable when not incorporated within supercomplex structures. Indeed, the complex III homodimer can remain intact when both mature complex I and the OXPHOS supercomplex are lost^23^.

Our BN-PAGE analysis detected MCAD in its ~175 kDa homotetramer and the ~450 kDa mitochondrial Hsp60 chaperonin complex^3^, as well as a number of other unknown complexes (Fig. 8). *In vitro* mitochondrial import assays revealed the presence of ~500 kDa and ~1,500 kDa MCAD-containing complexes, both of which reduced in intensity over the import time course. Conversely, SDS-PAGE analysis revealed that the intensity of imported, mature MCAD protein was similar over the time course. While these two complexes could be artefacts, their changing profiles in the face of constant MCAD protein levels would suggest that they are in fact *bona fide*, short-lived MCAD-containing complexes. Interestingly, the ~1,500 kDa complex is very close in size to the CI/CIII_2_ and CI/CIII_2_/CIV supercomplexes, and supports the previous description of MCAD and the OXPHOS supercomplex migrating together on BN-PAGE^10^.

BN-PAGE and Western blotting of HepG2 mitochondria with anti-MCAD antibodies also detected MCAD in unknown complexes of ~130 kDa and ~1,000 kDa. This ~1,000 kDa MCAD complex resolved at a similar size to OXPHOS complex I, however, it remained the same size when different detergents were used. Conversely, complex I resolved in various forms when solubilized in different detergents, suggesting that the ~1,000 kDa MCAD complex and complex I are in fact different complexes (and also that the ~1,000 kDa MCAD complex is not an integral membrane-bound complex). While the ~1,000 kDa MCAD-containing complex may be an artefact due to non-specific antibody binding, it is possible that MCAD may be part of a separate, large complex of ~1,000 kDa that does not contain OXPHOS complex I.

While the detection of these MCAD-containing complexes suggests physical interactions between MCAD and other mitochondrial proteins, our Co-IP and mass spectrometry analyses did not detect any specific protein-protein interactions with OXPHOS subunits. This may be due to the transient nature of any MCAD-OXPHOS protein interactions; indeed we were only able to detect short-lived MCAD-containing complexes by *in vitro* import assay. Furthermore, our BN-PAGE and Western blotting analysis suggests that the ~1,000 kDa MCAD-containing complex is not OXPHOS complex I. As such, it is possible that MCAD-OXPHOS interactions do not exist and that the OXPHOS instability and dysfunction that we have observed is due to another, as yet unidentified mechanism associated with the loss of MCAD protein.

Elevated oxidative stress has been suggested as a factor that contributes to the pathogenesis of MCAD deficiency, with increased levels of both oxidized proteins and phospholipids detected in MCAD-deficient patient samples^8,13^. Although we found a deficit in mitochondrial respiration in both MCAD-deficient patient cells and MCAD KO cells, we did not observe any increase in mitochondrial reactive oxygen species (ROS) generation under basal conditions. However, treatment with the OXPHOS complex III inhibitor antimycin A resulted in elevated mitochondrial ROS generation in MCAD KO cells compared to controls. Why this is the case is not clear, but it may be due to succinate-driven reverse electron transfer through the respiratory chain^28^. In control cells, blocking complex III will cause reverse electron flow, with ubiquinol oxidized and NAD^+^ reduced by complex I. However, in MCAD-deficient cells, NAD^+^ reduction is limited due to the reduced steady-state levels of complex I, resulting in the direct transfer of high-energy electrons to molecular oxygen to generate superoxide.

The loss of OXPHOS supercomplex stability in MCAD-deficient cells may also play a role in ROS generation. The formation of the OXPHOS supercomplex has been shown to limit the production of ROS^29^, whereas OXPHOS supercomplex destabilization has been shown to increase basal ROS generation in Barth Syndrome patient lymphoblasts^30^. The loss of the supercomplex structure in MCAD-deficient cells will also destabilize complex I, potentially exposing its flavin mononucleotide to molecular oxygen, resulting in increased ROS generation^31^. Whichever mechanism(s) is/are involved, it is apparent that any metabolic changes that alter electron transfer through OXPHOS complex III can result in elevated oxidative stress.

One important question that remains is how secondary OXPHOS defects may contribute to disease pathology in MCAD deficiency. Many individuals with MCAD deficiency are asymptomatic, suggesting that a secondary OXPHOS defect would have no influence on disease presentation. However, when clinical symptoms are triggered by a common illness or a period of fasting, a secondary defect in OXPHOS function may compound the primary defect in MCAD activity. This theory is supported by the outcomes of disease treatment, where encephalopathy or ventricular tachycardia can persist in patients, even though the prevailing hypoglycemia is corrected^20,32^. In these instances, it may be hypothesized that treatment efficacy is reduced due to the secondary defect in OXPHOS function and glucose metabolism. These acute effects may also be compounded by an increase in oxidative stress, particularly by disruption of electron transfer through OXPHOS complex III due to environmental and/or genetic effects.

In summary, our results describe a new role for MCAD in OXPHOS complex biogenesis. While it remains to be determined exactly how MCAD interacts with the OXPHOS complexes and/or the OXPHOS supercomplex, it is clear that the loss of MCAD has a detrimental effect on OXPHOS complex stability and activity. These findings have implications for our understanding of MCAD deficiency, where OXPHOS dysfunction may contribute to disease pathogenesis.

## Methods

### Patient Details

Patient 1 is a female of Japanese descent who presented at 15 months of age with a hypoglycemic crisis consisting of vomiting and disturbed consciousness (seizure, drowsiness and apnea). Newborn screening for MCAD deficiency was not available in Japan at the time of birth. Retrospective analysis of her stored newborn blood spot revealed C8-acylcarnitine levels of 2.52 nmol/mL (normal range < 0. 3), while MCAD activity in lymphocytes was 1.5 pmol/min/10^6^ cells (control activity is 47.5 ± 13.2) [Referred to as Patient 4 in^33^]. At 22 months of age during an episode of drowsiness with persistent apnea, blood free fatty acid levels were: C8, 388.0 nmol/mL (control range 2.3–8.7); C10, 19.3 nmol/mL (control range 2.8–7.1), C10:1, 90.0 nmol/mL (control range < 2 nmol/mL). At 25 months of age (with persistent apena only), blood free fatty acid levels were: C8, 32.3 nmol/mL (control range 2.3–8.7); C10, 5.0 nmol/mL (control range 2.8–7.1), C10:1, 18.6 nmol/mL (control range < 2 nmol/mL). Genetic analysis revealed a 4.9 kB deletion of the paternal *ACADM* allele, which includes exons 11 and 12, while a large deletion including the whole *ACADM* gene was identified in the maternal allele by fluorescence *in situ* hybridization.

Patient 2 is a healthy female of Japanese descent who was identified as having MCAD deficiency by tandem mass spectrometry newborn screening. Analysis of her dried blood spot at 5 days of age revealed C8-acylcarnitine levels of 0.44 nmol/mL (normal range < 0.3) and a C8/C10 acylcarnitine ratio of 1.81 (normal range < 1.4). MCAD activity in lymphocytes was 6.9 pmol/min/10^6^ cells (control activity is 47.5 ± 13.2). Genetic analysis revealed a homozygous *ACADM* mutation, c.1085G>A (p.G362E precursor form; p.G337E mature form), which has been previously reported^34^. Life-threatening attacks and complications including hypoglycemia, liver disorder and muscle pain have not been reported.

All experiments using patient fibroblasts were performed in accordance with relevant guidelines and regulations, with human ethics approved by the Chiba Children’s Hospital Ethics Committee (No. 20150701), the Saitama Medical University Ethics Committee (No. 482) and the Monash University Human Research Ethics Committee (No. CF12/2490–2012001345). Informed consent was obtained from all participants and/or their legal guardians.

### Cell culture

Control fibroblasts (passage 12 to 16), Patient 1 fibroblasts (passage 5 to 7) and Patient 2 fibroblasts (passage 3 to 5) were cultured at 37 °C/5% CO_2_ in DMEM supplemented with 10% (v/v) fetal calf serum (FCS), 1x penicillin/streptomycin, 50 µg/mL uridine and 1x GlutaMAX^TM^ (Thermo Fisher Scientific, Waltham, MA, USA). 143BTK^−^ osteosarcoma cells (143B cells) and 143B MCAD ‘knockout’ cells (matched passages 25 to 30), HEK293T cells and HepG2 cells were cultured under similar conditions but in the presence of 5% (v/v) FCS.

### Generation of MCAD ‘knockout’ cells by CRISPR/Cas9 gene editing

An oligonucleotide pair targeting exon 2 of the *ACADM* gene was phosphorylated, annealed and ligated into the lentiCrisprV2 vector containing hSpCas9 and a chimeric guide RNA^35^. The resulting vector was transfected with the packaging vectors pVSVg and psPAX2 into HEK293T cells using Lipofectamine 2000 (Thermo Fisher Scientific). After 48 h, the virus-containing media was passed through a 0.45 µm filter and supplemented with 5 µg/mL polybrene (Sigma, St. Louis, MO, USA) before being added to 143B cells. Transformed cells were selected with 1.0 µg/mL puromycin and single-cell colonies generated. Loss of MCAD protein expression was confirmed for each clone by Western blotting, while genotype was determined by PCR and Sanger sequencing.

### Measurement of oxygen consumption rates

Oxygen consumption rates (OCR) of patient-derived skin fibroblasts were analyzed by microscale respirometry with the XF96 extracellular flux analyser (Seahorse XF96; Seahorse Bioscience, Billerica, MA, USA) according to^8,13^. Control and patient fibroblasts were seeded in a 96-well plate at 2 × 10^4^ cells/well in 80 μL of growth medium and incubated for 24 h at 37 °C/5% CO_2_. Media was replaced with 160 μL of unbuffered DMEM containing 25 mM glucose, 1 mM sodium pyruvate and 2 mM glutamine, followed by incubation at 37 °C without CO_2_ for 1 h. Following the measurement of basal OCR, oligomycin (2 μM final), carbonyl cyanide 4-(trifluoromethoxy)phenylhydrazone (FCCP, 0.4 μM final) and rotenone (1 μM final) were injected sequentially and OCR measured for 3 min following each injection. OCR were expressed as pmol/min/20,000 cells, normalized by cell number using the CyQUANT Cell proliferation kit (Invitrogen). FCCP-stimulated rates were calculated minus rotenone (which was used to detect the presence of mitochondrial respiration that is sensitive to a respiratory inhibitor) and expressed relative to controls. Student’s two-tailed *t*-test was used for statistical analysis.

High-resolution respirometry using 143B cells was performed with an Oxygraph-2K oxygen electrode (Oroboros, Innsbruck, Austria). Respiration was measured in either DMEM (25 mM glucose) with 10% (v/v) FCS, 1x penicillin/streptomycin, 50 µg/mL uridine and 1x GlutaMAX^TM^ or DMEM (no glucose) with 10% (v/v) dialyzed FCS, 10 mM galactose, 50 µg/mL uridine, 1x GlutaMAX™ and 2 mM L-glutamine. Uncoupled, maximal respiration was measured in the presence of 5 µM FCCP. Mitochondrial respiration rates were calculated using DatLab software (version 4.3.4.51, Oroboros Instruments) and expressed as pmol O_2_/s/mg whole cell protein. Experiments were performed three times with significant differences determined using Student’s two-tailed *t*-test.

### Mitochondrial isolation

Cell pellets were resuspended in Solution A [20 mM HEPES pH 7.6, 220 mM mannitol, 70 mM sucrose, 1 mM EDTA, 0.5 mM phenylmethylsulfonyl fluoride (PMSF), 2 mg/mL fatty acid-free BSA] and incubated on ice for 15 min. Cells were homogenized with 20 strokes of a drill-fitted Teflon pestle, then spun at 800 *g* for 10 min, 4 °C to pellet cell debris and nuclei. The supernatant was removed and spun at 10,000 *g* for 20 min, 4 °C. The resulting mitochondrial pellets were washed in Solution B (Solution A without BSA), then resuspended in Solution B for downstream analysis. Mitochondrial protein quantitation was determined as described^36^.

### Blue-native polyacrylamide gel electrophoresis (BN-PAGE)

BN-PAGE was performed as previously described^37^. In brief, 40 μg of isolated mitochondria were solubilised for 30 min on ice in 50 μL of 1% (v/v) Triton X-100 (Sigma) or 1% (w/v) digitonin (Merck, NJ, USA). Samples were spun at 16,000 *g* for 5 min, 4 °C to pellet insoluble material, and the supernatant was combined with 5 μL of 10x BN-PAGE loading dye [5% (w/v) Coomassie Blue G, 500 mM Ɛ-amino-n-caproic acid]. Samples were resolved on either a 4–10% (w/v) or 4–13% (w/v) BN-PAGE gel at 100 V/5 mA for approximately 14 h at 4 °C.

### Denaturing gel electrophoresis and 2-dimensional (2D) native-PAGE

Mitochondrial protein was separated using denaturing gel electrophoresis as previously described^37^. In brief, isolated mitochondria were solubilised in sample buffer [50 mM Tris-Cl, pH 6.8, 100 mM DTT, 2% (w/v) SDS, 10% (v/v) glycerol, 0.05% (w/v) bromophenol blue] before separation on a 12–18% (w/v) Tris-tricine continuous gradient gel (50 µg of mitochondrial protein) or a 10% (w/v) SDS-polyacrylamide gel (20 µg of mitochondrial protein).

For 2D native-PAGE, 80 µg of mitochondrial protein was first resolved on a 4–10% (w/v) BN-PAGE gel. Gel slices were then excised for second dimension separation using a 12–18% (w/v) Tris-tricine gel^37^.

### Western blotting

Western blotting was performed as previously described^38^. Proteins were transferred to PVDF membrane using a semi-dry method, blocked with 10% (w/v) skim-milk in 1x PBS / 0.05% (v/v) Tween-20, then probed overnight at 4 °C with primary antibodies. Membranes were subsequently incubated with the appropriate horseradish peroxidase (HRP)-coupled secondary antibody, and proteins visualized with ECL (GE Healthcare, Little Chalfont, Buckinghamshire, UK) using a Microchemi 4.2 Gel Imaging System (DNR Bio-imaging Systems, Jerusalem, Israel) with a 16-bit (65,536 levels of grey) CCD camera.

Primary antibodies used were against MCAD (Abcam, Cambridge, UK, ab110296) and subunits of the OXPHOS complexes (‘Mitoprofile’ total OXPHOS Antibody Cocktail, Abcam, ab110411). For BN-PAGE, antibodies were used to detected the TOM complex (TOMM40, Santa Cruz Biotechnology, SC-11414) OXPHOS complex II (SDHA, Abcam, ab14715), complex V (ATP5A, Abcam, ab14748), complex III and the III_2_/IV supercomplex (UQCRC1, Abcam, ab110252), complex IV (COI, Abcam, ab14705) and complex I and the I/III_2_/IV and I/III_2_ supercomplexes (NDUFA9, raised in rabbits as previously described^39^).

Protein band intensities were calculated using ImageJ software (NIH) from at least three independent experiments. Regions of Interest (ROIs) containing protein complex signals were selected from non-saturated images (signal intensity < 65,536) and background signals (selected using an identical ROI as for the primary signal) subtracted. Protein complex levels were standardized by dividing by OXPHOS complex II levels as a loading control, resulting in a ratio which was then converted to % of control levels: Patient 1 relative to Control 1; Patient 2 relative to Control 2; 143B MCAD KO relative to 143B Control (Note that Complex II steady-state levels in Patient mitochondria were 83.5 ± 32.0% of Control mitochondria steady-state levels overall). Averages, standard deviation and Student’s two-tailed *t*-tests to determine significant differences were calculated using Microsoft Excel. All quantitation for Western blotting in Figs 1 and 2 are provided in Supplemental Table S2. Full Western blot images are shown in Supplemental Fig. 3.

### Radiolabeling of mtDNA-encoded proteins

MtDNA-encoded proteins were radiolabeled with [^35^S]-methionine/cysteine as described previously^37^, but without chloramphenicol pre-treatment. Cells were grown in 10 cm dishes and incubated in 6 mL of methionine-free DMEM containing 5% (v/v) dialyzed FCS and 0.1 mg/mL cycloheximide for 15 min at 37 °C/5% CO_2_. Proteins were radiolabeled by adding 7 µL of [^35^S]-methionine/cysteine (11 mCi/mL; EXPRE^35^S^35^S Protein Labelling Mix; Perkin Elmer Life Sciences, Waltham, MA, USA) and incubated for 2 h at 37 °C. Unlabeled methionine was then added to a final concentration of 0.1 mM and cells were incubated for 15 min at 37 °C. The labelling media was replaced with DMEM supplemented with 10% (v/v) FCS, 1x penicillin/streptomycin, 50 µg/mL uridine and 1x GlutaMAX^TM^ for chase times of 0, 3 and 24 h. Mitochondria were isolated and separated by SDS-PAGE, BN-PAGE or 2D native-PAGE, with proteins transferred to PVDF membranes before exposure to storage phosphor screens (GE Healthcare) and detection using a Typhoon Laser Scanner (GE Healthcare). Protein band intensities were calculated using ImageJ (NIH) software from at least three independent experiments. Regions of Interest (ROIs) containing protein complex signals were selected and background signals (selected using an identical ROI for the primary signal) subtracted. Band intensities were converted to % of control levels (143B MCAD KO relative to 143B Control). For 2D-PAGE quantitation, intensities of individual supercomplex proteins were standardized to ATP6 signal intensities. Averages, standard deviation and Student’s two-tailed *t*-tests to determine significant differences were calculated using Microsoft Excel. All quantitation for data shown in Figs 4 and 5 are provided in Supplemental Table S3.

### *In vitro* mitochondrial import assays

CDNAs encoding MCAD, NDUFA9 and COX VIa-L (liver isoform) were cloned into the pGEM4Z vector (Promega, Madison, WI, USA) and proteins translated using the TnT Coupled Reticulocyte Lysate System (Promega) in the presence of [^35^S]-methionine/cysteine. Translation products were incubated with freshly isolated mitochondria in 250 mM sucrose, 80 mM potassium acetate, 5 mM magnesium acetate, 10 mM sodium succinate, 1 mM dithiothreitol, 5 mM ATP and 20 mM HEPES pH 7.4 at 37 °C for the times indicated. Dissipation of the mitochondrial membrane potential (Δψ_m_) was performed in the presence of 10 µM FCCP (with no ATP or sodium succinate). Samples subjected to protease treatment were incubated on ice for 10 min with 100 μg/mL proteinase K (Sigma) before treatment with 1 mM PMSF for 10 min.

Protein band intensities were calculated using ImageJ (NIH) software from at least three independent experiments. Regions of Interest (ROIs) containing protein complex signals were selected and background signals (selected using an identical ROI for the primary signal) subtracted. Band intensities were converted to % of control levels (143B MCAD KO relative to 143B Control). For MCAD import experiments (Fig. 8), band intensities were standardized to the ~450 kDa mitochondrial chaperonin complex signal. Averages, standard deviation and Student’s two-tailed *t*-tests to determine significant differences were calculated using Microsoft Excel. All quantitation for data shown in Figs 6, 7 and 8 are provided in Supplemental Table S3.

### Measurement of mitochondrial superoxide generation

Cells were incubated with 2 μg/mL Hoechst 33342 (Thermo Fisher Scientific, H1399) and 3 μM MitoSOX^TM^ (Thermo Fisher Scientific, M36008) in DMEM supplemented with 5% (v/v) FCS, 1x penicillin/streptomycin, 50 µg/mL uridine and 1x GlutaMAX^TM^ for 20 min. Cells were washed with phenol red-free DMEM (Thermo Fisher Scientific, 21063-029) before incubation in phenol red-free DMEM supplemented with 5% (v/v) FCS, 1x penicillin/streptomycin, 50 µg/mL uridine and 1x sodium pyruvate (Thermo Fisher Scientific), with or without 2 µM rotenone (Sigma) or 4 µM antimycin A (Sigma). Cells were imaged every 15 min using an ArrayScan Vti High Content Analyser (Thermo Scientific, MA, USA), with Hoechst 33342 and MitoSOX^TM^ fluorescence excited with 365 nm and 549 nm laser lines respectively. Fluorescence measurements were averaged from five wells containing approximately 100 cells per well. Significant differences between treated and untreated control and MCAD ‘knockout’ cells were determined by ANOVA with Tukey’s post-hoc multiple comparison tests.

### Co-immunoprecipitation and mass-spectrometry

Co-immunoprecipitation (Co-IP) was performed using mitochondria isolated from either 143B control cells, 143B MCAD ‘knockout’ cells or HepG2 cells. Approximately 1 mg of mitochondria was resuspended in 1 mL of solubilisation buffer [100 mM NaCl, 20 mM Tris-HCl pH 7.4 with either 1% (v/v) Triton X-100 or 1% (w/v) digitonin] for 30 min on ice. Insoluble material was pelleted at 21,000 *g*, with the supernatant incubated with Protein A-sepharose (Sigma) alone (negative control) or Protein A-sepharose cross-linked with anti-MCAD antibodies at 4 °C overnight. The Protein A-sepharose beads were washed three times in solubilisation buffer, followed by one wash in pre-elution buffer [10 mM NaCl, 2 mM Tris-HCl pH 7.4 with either 0.1% (v/v) Triton X-100 or 0.1% (w/v) digitonin]. Proteins were eluted in 0.1 M Glycine pH 2.5, acetone precipitated, reduced, alkylated, trypsin digested and desalted as previously described^40^. Peptides were reconstituted in 0.1% (v/v) trifluoroacetic acid (TFA) and 2% (v/v) acetonitrile (ACN) and analyzed by online nano-HPLC/electrospray ionization-MS/MS on a Q Exactive Plus connected to an Ultimate 3000 HPLC (Thermo-Fisher Scientific). Peptides were loaded onto a trap column (Acclaim C_18_ PepMap nano Trap × 2 cm, 100 μm I.D, 5 μm particle size and 300 Å pore size; ThermoFisher Scientific) at 15 μL/min for 3 min before switching the pre-column in line with the analytical column (Acclaim RSLC C_18_ PepMap Acclaim RSLC nanocolumn 75 μm × 50 cm, PepMap100 C_18_, 3 μm particle size 100 Å pore size; ThermoFisher Scientific). The separation of peptides was performed at 250 nL/min using a linear ACN gradient of buffer A [0.1% (v/v) formic acid, 2% (v/v) ACN] and buffer B [0.1% (v/v) formic acid, 80% (v/v) ACN], starting at 12.5% buffer B to 38.5% followed by ramp to 99% over 30 min. Data were collected in positive mode using Data Dependent Acquisition using m/z 375–1800 as MS scan range, HCD for MS/MS of the 12 most intense ions with z ≥ 2. Other instrument parameters were: MS1 scan at 70,000 resolution (at 200 m/z), MS maximum injection time 50 ms, AGC target 3E6, Normalized collision energy was at 27% energy, Isolation window of 1.8 Da, MS/MS resolution 17,500, MS/MS AGC target of 1E5, MS/MS maximum injection time 100 ms, minimum intensity was set at 1E3 and dynamic exclusion was set to 20 sec. Raw files were analyzed using the MaxQuant platform^41^ version 1.5.8.0 searching against the Uniprot human database containing reviewed, canonical and isoform variants in FASTA format (June 2016) and a database containing common contaminants. Default search parameters for a label-free (LFQ) experiment were used. Briefly, multiplicity was set to 1 (unlabeled), “LFQ”, “Re-quantify” and “Match between runs” were enabled with default settings. Unique and razor peptides were used for quantification, using a minimum ratio count of 2. Using the Perseus platform^42^ version 1.5.5.3, proteins with a sequence coverage of <5%, or identified using <2 unique peptides were excluded. Missing values were imputed to values based on the distribution of the total matrix with downshift of 1.8 and width of 0.3. Mitochondrial proteins were defined through matching of gene names to the Mitocarta2.0 dataset^43^. A modified two-sided *t*-test based on permutation-based FDR statistics^42^ was performed (FDR < 0.01, S0 = 1) between experimental groups and the negative logarithmic *p-*values were plotted against the differences between the Log_2_ means for the two groups. Raw data, including q-values used for indicating significance, can be found in Supplemental Table S1.

## Electronic supplementary material

Supplemental Information

Supplementary Dataset 1

Supplementary Dataset 2

Supplementary Dataset 3
