# Supplementary material for: Loss of the Mitochondrial Fatty Acid β-Oxidation Protein Medium-Chain Acyl-Coenzyme A Dehydrogenase Disrupts Oxidative Phosphorylation Protein Complex Stability and Function
Source: Sci Rep. 2018 Jan 9;8:153. doi: 10.1038/s41598-017-18530-4 (PMC5760697; doi:10.1038/s41598-017-18530-4)
Supplement: Supplementary file 1 — Supplemental Information [file 41598_2017_18530_MOESM1_ESM.pdf]

Loss of the Mitochondrial Fatty Acid β-Oxidation Protein Medium-Chain Acyl-Coenzyme A Dehydrogenase Disrupts Oxidative Phosphorylation Protein Complex Stability and Function

Sze Chern Lim[1,2], Makiko Tajika[3], Masaru Shimura[3], Kirstyn T. Carey[4], David A. Stroud[5], Kei Murayama[3], Akira Ohtake[6] and *Matthew McKenzie[1,2].

Supplemental Figures and Information

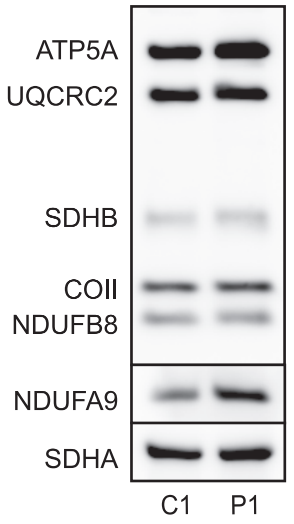

Supplemental Figure 1. Steady-state levels of OXPHOS complex subunits.

Mitochondria were isolated from control (C1) or MCAD-deficient patient (P1) fibroblasts for SDS-PAGE and Western blot analysis. The steady-state levels of NDUFB8 (complex I), SDHA and SDHB (complex II), UQCRC2 (complex III), COII (complex IV) and ATP5A (complex V) in P1 patient mitochondria were not different to the control. Levels of the complex I subunit NDUFA9 appear higher in P1 mitochondria but were not significantly different across multiple experiments (121 ± 23%, p=0.19, n=3).

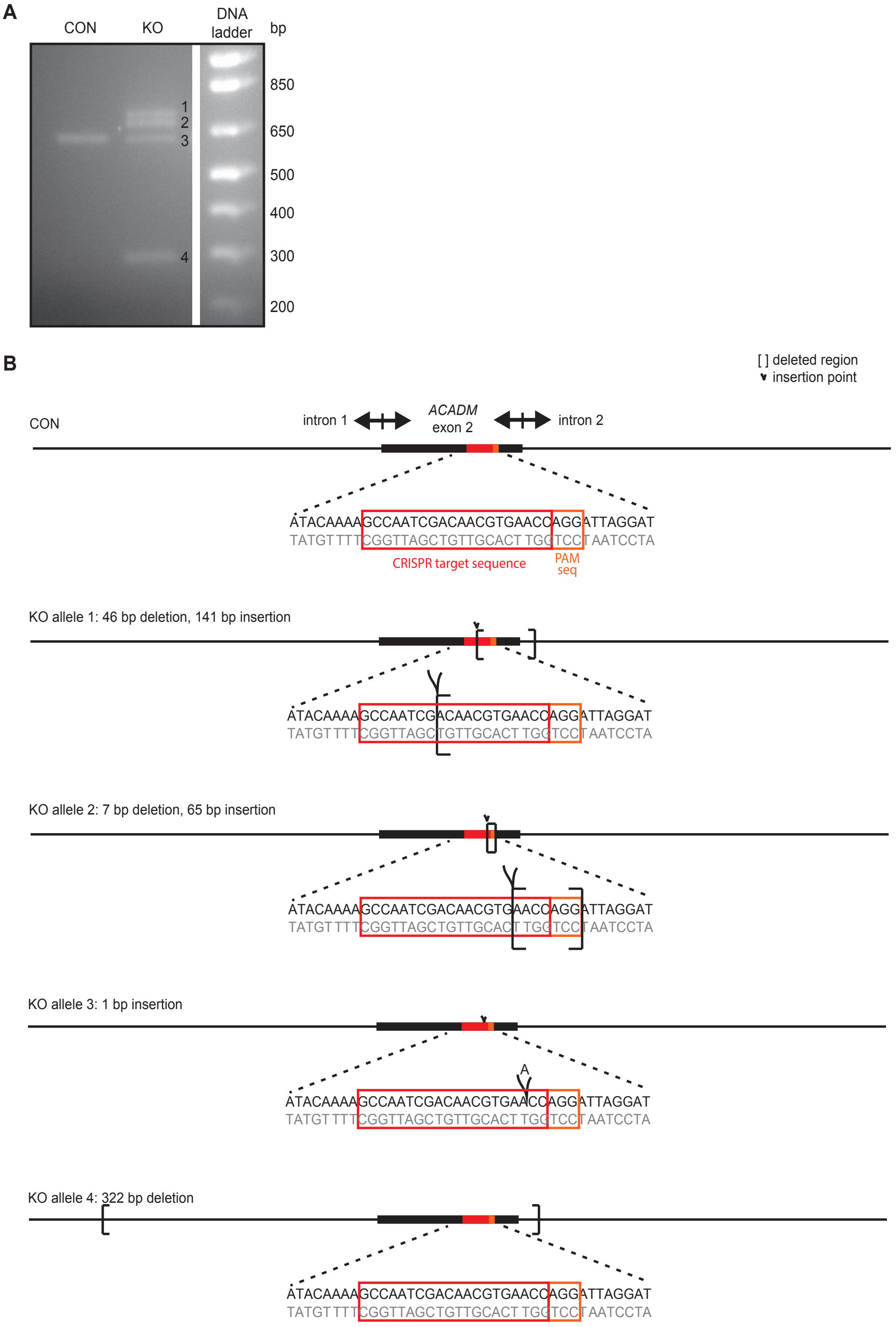

Supplemental Figure 2. Generation of 143B MCAD knockout cells using CRISPR/Cas9.

(A) Gel electrophoresis showing PCR amplicons containing exon 2 of the *ACADM* gene from gDNA of 143B control (CON) and 143B MCAD 'knockout' (KO) cells (clone number 2). Four different PCR amplicons (1 to 4) were generated from MCAD KO cells. **(B)** Illustration of *ACADM* showing exon 2, introns 1 and 2, the CRISPR target sequence (red) and PAM sequence (orange). Four alleles were mutated in the KO cell line: allele 1 has a 46 bp deletion in exon 2 and intron 2, and a 141 bp insertion in the same region; allele 2 has a 7 bp deletion of the CRISPR target sequence and PAM sequence, and a 65 bp insertion in the same region; allele 3 has a 1 bp insertion in the CRISPR target sequence; allele 4 has a 322 bp deletion of the entire exon 2 and parts of introns 1and 2.

Supplemental Figure 3. Complete Western blot images from Figure 1 and Figure 2.

Figure 1

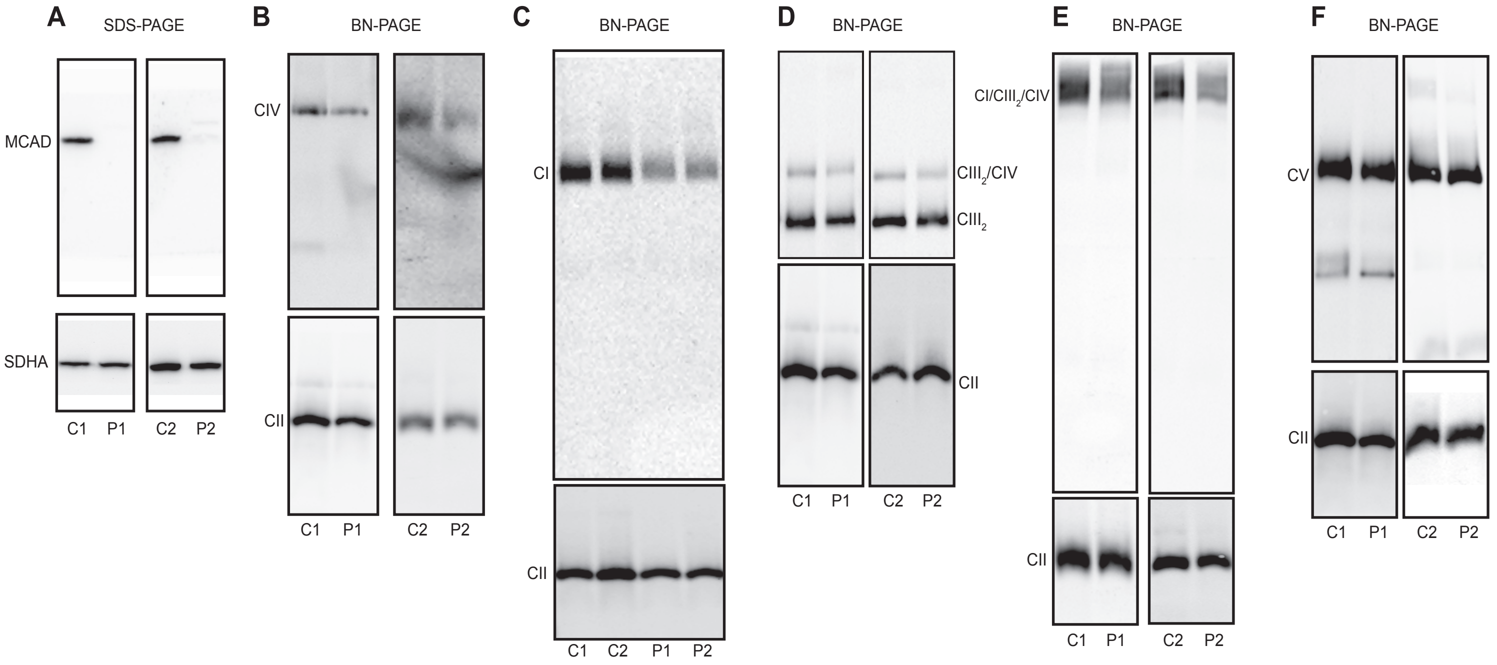

Figure 2

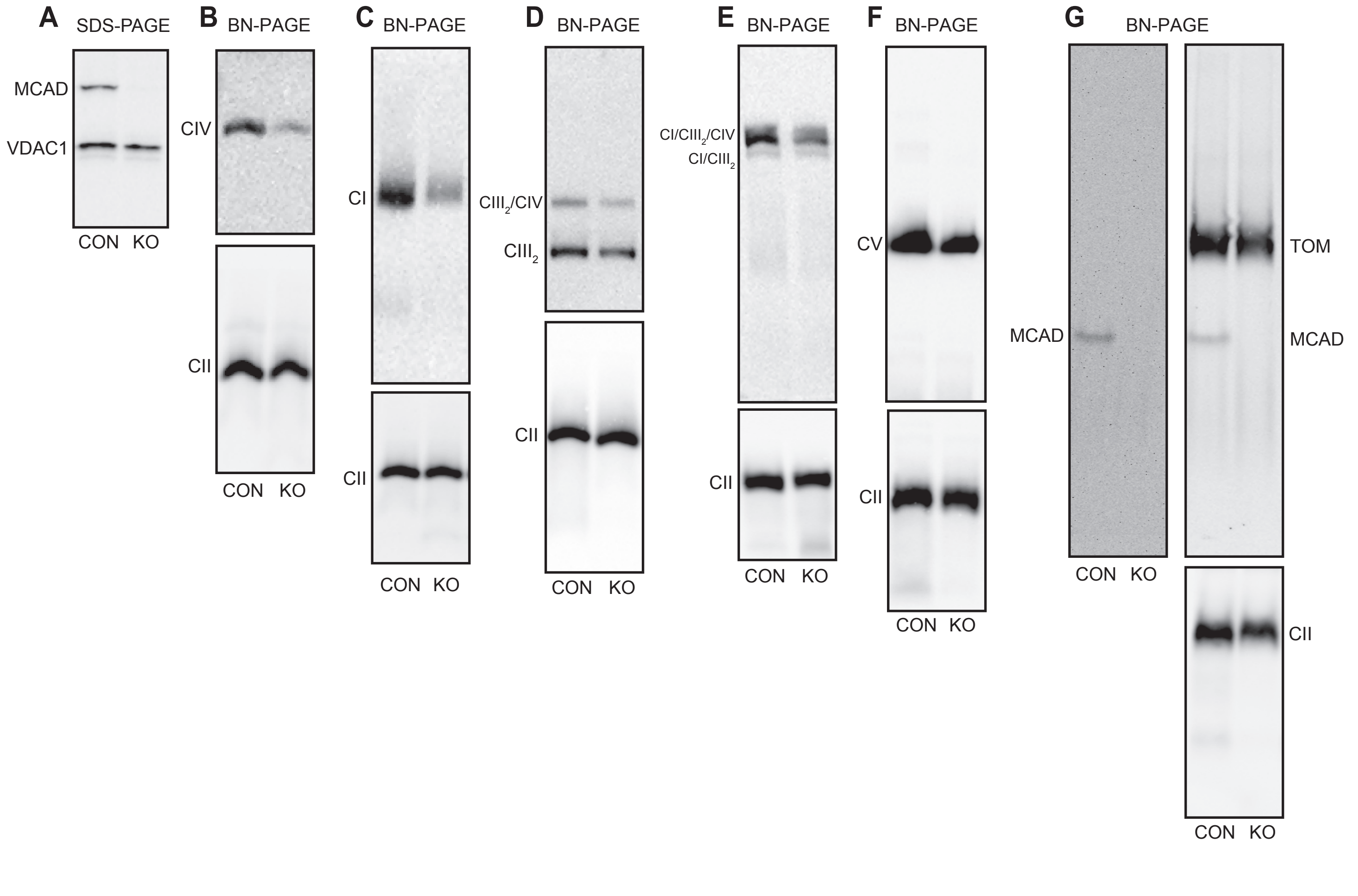

Supplemental Table S1. Sortable results from co-immunoprecipitation and mass-spectrometry analyses in Figure 9. Difference Log_2 LFQ intensity, *t*-test p-value, q-value and raw Log_2 LFQ intensities for each experiment. Results are ordered according to the Log_2 difference in Figure 9A by default.

Supplemental Table S2. BN-PAGE Western blotting Quantitation for Figures 1 & 2.

Supplemental Table S3. Quantitation for Figures 4, 5, 6, 7 and 8.
